# Pre-clerkship clinical skills and clinical reasoning course performance: Explaining the variance in clerkship performance: Pre-clerkship predictors of clerkship variance

**DOI:** 10.1007/s40037-016-0287-z

**Published:** 2016-07-18

**Authors:** Jeffrey S. LaRochelle, Ting Dong, Steven J. Durning

**Affiliations:** Department of Medicine, Uniformed Services University of the Health Sciences, Bethesda, MD USA

**Keywords:** Clinical skills, Clinical reasoning, Struggling learners

## Abstract

Introduction: Evidence suggests that pre-clerkship courses in clinical skills and clinical reasoning positively impact student performance on the clerkship. Given the increasing emphasis on reducing diagnostic reasoning errors, it is very important to develop this critical area of medical education. An integrated approach between clinical skills and clinical reasoning courses may better predict struggling learners, and better allocate scarce resources to remediate these learners before the clerkship. Methods: Pre-clerkship and clerkship outcome measures from 514 medical students graduating between 2009 and 2011were analyzed in a multiple linear regression model. Results: Learners with poor performances on integrated pre-clerkship outcome measures had a relative risk of 6.96 and 5.85 for poor performance on National Board of Medical Examiners (NBME) subject exams and clerkship performance, respectively, and explained 22 % of the variance in clerkship NBME subject exam scores and 20.2 % of the variance in clerkship grades. Discussion: Pre-clerkship outcome measures from clinical skills and clinical reasoning courses explained a significant amount of clerkship performance beyond baseline academic ability. These courses provide valuable information regarding student abilities, and may serve as an early indicator for students requiring remediation. Conclusions: Integrating pre-clerkship outcome measures may be an important aspect of ensuring the validity of this information as the pre-clerkship curriculum becomes compressed, and may serve as the basis for identifying students in need of clinical skills remediation.

## What this paper adds

- The development of basic clinical skills prior to students entering the clerkship is critically important, but identifying those students who may be at risk of poor performance in clinical skills or clinical reasoning on the clerkship can be challenging.
- Research has demonstrated the benefit of longitudinal assessments in identifying deficits in knowledge.
- We found that using integrated clinical skills and clinical reasoning assessments identifies students at risk of struggling in these domains at the clerkship level, and provides an opportunity for early remediation of these learners prior to the start of the clerkship.

## Introduction

Although many medical schools in the United States continue to adhere to a predominantly ‘Flexnerian’ model (two years of basic science courses followed by two years of clinical rotations), several medical schools are undergoing significant curriculum reforms aimed at integrating basic sciences with clinical skills teaching while often reducing the pre-clerkship period to facilitate an early transition to the clinical clerkships [1]. Despite the compression of the pre-clerkship training period, students tend to want more advanced clinical skills training in preparation for their clerkship experience, while faculty remain focused on the basic components of communication skills, patient interviewing, physical exam skills, and clinical reasoning as the foundation for being an effective physician [1, 2]. However, there remains limited consensus on how to deliver pre-clerkship education and, furthermore, many clerkship directors continue to feel that students are not adequately prepared for the clerkship [3, 4].

Regardless, pre-clerkship courses in clinical skills and clinical reasoning have been shown to improve student performance during the clerkships in foundational skills such as communication and development of patient relationships, in addition to student motivational aspects such as educational attitudes, initiative, participation, and dependability [5]. Although isolated pre-clerkship outcomes, such as an objective structured clinical examination (OSCE), are associated with failures on a subsequent OSCE, individual pre-clerkship measures often do not adequately assess the overall impact of pre-clerkship teaching on student clinical performance in the clerkship [5–7]. Some have suggested that a combination of pre-clerkship assessments may better explain the variance in a student’s clinical abilities on the clerkship [3].

Previous studies have consistently demonstrated that performance on standardized exams is correlated to performance on future standardized exams [8]. Further, the implementation of longitudinal progress testing is able to identify learners with persistent knowledge deficits and reduce licensing exam failures [9, 10]. Since the observation and remediation of medical students’ clinical skills on the clerkship is inconsistent, the early identification of students who may struggle in the clerkship period would be of significant benefit [11, 12]. The pre-clerkship period offers the opportunity for the deliberate practice of clinical skills in a controlled environment (e. g. a simulation centre using standardized patients), which has been found to be a better approach for the remediation of clinical skills deficits [13]. However, the ability to identify students with persistent clinical skills or clinical reasoning deficits in the pre-clerkship period that may persist in the clerkship has proven to be challenging [7, 8]. The integration of the pre-clerkship courses, Introduction to Clinical Medicine (ICM) and Introduction to Clinical Reasoning (ICR), at our institution provided a unique opportunity to examine how integrated pre-clerkship assessments may better explain the variance of student performance in the clerkships, and identify students likely to struggle in the clerkship.

Prior to a comprehensive curriculum re-design at our institution, the leadership of the ICR and ICM courses began to work towards integration in both instruction and evaluation of learners. Previously, the ICM course was primarily focused on teaching and evaluating clinical skills (communication, history taking, and physical exam) while the ICR course was primarily concerned with the development of clinical reasoning, and both courses functioned independently using separate cases and evaluations. The first step towards integration was to develop joint evaluations on the end of pre-clerkship OSCE, in addition to including an oral case presentation as a synthetic evaluation of clinical reasoning within each standardized patient encounter for the ICS course. Additionally, we moved towards integrating course content by using the standardized patient encounters in ICS as the cases for clinical reasoning discussion within the ICR course. Consistent with situativity theory, our goal for integration was to bring together the context and environment of a clinical encounter using a standardized patient so that students were active participants in the learning of both clinical skills and clinical reasoning similar to what they would experience on the clerkship [14]. Even in the early stages of integration at the time of this study, our focus was on the application of skills and knowledge to improve clinical reasoning, and given the emphasis on reducing diagnostic reasoning errors through improvements in health professions education, the early identification of struggling learners represents a significant opportunity for educational interventions [15, 16].

Our study was designed to examine the extent that integrated pre-clerkship course evaluations from ICM and ICR are associated with students’ clinical and standardized test performance across all their clerkships. We hypothesized that these integrated pre-clerkship outcomes would not only demonstrate a strong association with clinical performance on the clerkships and potentially identify at risk students, but also explain a significant amount of the variance in their performance independent of baseline academic abilities.

## Methods

### Study context and participants

This investigation was part of the larger Long-Term Career Outcome Study (LTCOS) conducted at the F. Edward Hébert School of Medicine, Uniformed Services University (USU), and was granted ethical approval through the USU Institutional Review Board. As the United States’ only federal medical school, USU matriculates approximately 170 medical students annually and, at the time of this study, offered a traditional four-year curriculum: two years of basic science courses followed by two years of clinical rotations (clerkships). Both the ICM and ICR courses run throughout the entire second year of medical school. The ICM course is a case-based curriculum that uses standardized patient encounters with direct faculty observation to teach basic clinical skills. The ICR course is also a case-based curriculum that uses a combination of didactics and small-group teaching to deliver instruction on a broad variety of clinical reasoning techniques [17]. The participants of the present study were students graduating in 2009 through 2011 (*n* = 514; 143 were female (27.8 %) and 371 were male (72.2 %)).

### Measures and statistical analysis

#### ICM course performance

Measures of student performance in the ICM course included a preceptor evaluation, an OSCE, and the National Board of Medical Examiners (NBME) subject exam on the Introduction to Clinical Diagnosis (ICD). The preceptor evaluations are based on direct observation of basic clinical skills (history taking, physical examination, oral case presentation, and written notes) over six sessions at the National Capital Area Simulation Center using standardized patient encounters. The OSCE and the ICD NBME subject exam are integrated assessments with ICR, and are described below.

#### ICR course performance

The students’ performance in the ICR course was measured on faculty-derived exam points, which consists of two multiple choice examinations and one cumulative essay examination. Additionally, students receive points based upon their small-group discussions, where students are graded according to their level of participation in each of over 30 small-group sessions that deal with common symptoms, findings, and syndromes in medicine using a case-based approach.

#### Integrated ICM and ICR assessments

The OSCE is a six-station exam evaluating basic clinical exam skills (communication skills, history taking, physical exam skills, and medical knowledge) and clinical reasoning across multiple content domains to include neurology, geriatrics, gastroenterology, endocrinology and anaemia given at the end of the pre-clerkship period. Learners are assessed by a combination of checklists completed by trained standardized patients, multiple choice items, and free text responses evaluated by an experienced clinician educator. Additionally, there is an oral case presentation station where learners accomplish a face-to-face presentation with an experienced clinician educator, who uses a standard form to evaluate learners’ performance. According to a previously conducted internal quality improvement study, a generalizability study for the pre-clerkship OSCE demonstrated a moderate generalizability coefficient (r = 0.52) with 78.1 % of the variance in scores attributable to student variables. The ICD NBME subject exam is also a shared assessment between the ICM and ICR course at the end of the pre-clerkship period, and covers basic clinical reasoning and diagnosis across all organ systems.

#### NBME score across clerkships

The NBME offers a variety of multiple-choice clinically specific subject exams for medical students during their clerkship year. NBME subject exams for individual clerkships cover more advanced clinical reasoning and diagnosis skills in addition to applying the principles of patient management. During the study period, students completed subject exams in obstetrics and gynaecology, paediatrics, family medicine, general surgery, psychiatry and internal medicine at the conclusion of each individual clerkship, respectively.

#### Clerkship final grade

Final clerkship grades were included as an additional marker of student clinical performance. Although there is some variability in how final grades are determined across clerkships, the major component in all the clerkship grades is direct faculty observation of learner performance in the context of patient care. In addition, individual clerkships utilize the NBME subject exams and formal clinical skills assessments (e. g. OSCE or task trainers) to determine final student grades. In the context of this study, we converted these final grades (A, B, C, D/F) into the nominal variables of 1, 2, 3, or 4 for the purposes of statistical analysis.

### Statistical analysis

We first reported the descriptive statistics of all the course assessments and clerkship outcome measures. To examine the students’ ICM and ICR course assessments predictive power on average clerkship NBME subject exam score as well as average final grade across all six clerkships, we developed a multiple linear regression model. The outcome variables for the regression analysis were the average clerkship NBME subject exam score across six clerkships and the average clerkship final grade. The first-year grade point average was entered in the regression model as a control variable, and the pre-clerkship outcomes were entered next in one block. The purpose was to see how much additional variance the pre-clerkship outcome measures could explain beyond the first year grade point average. In addition, we identified at risk, defined as students with overall course scores greater than 1.5 standard deviations below the class mean (bottom 13^th^ percentile), in both the ICM and ICR course, and determined the relative risk of clerkship NBME subject exam failures and/or less than passing final clerkship grades. All the statistical analyses were conducted using SPSS 22.0.

## Results

Students identified as at risk in either the ICM or ICR course were found to have significantly lower scores in both average clerkship grades (2.75 ± 0.36 v. 3.22 ± 0.39, *p* < 0.001) and on average clerkship NBME scores (69.0 ± 4.61 v. 73.0 ± 5.52, *p* < 0.001). The effect size as measured by a Cohen’s d was large for both the impact on average clerkship grades (d = 1.25) and average clerkship NBME scores (d = 0.79). In addition, students at risk in both the clinical skills and clinical reasoning courses had a relative risk of 5.85 (95 % CI of 1.88–18.2, *p* = 0.002) and 6.96 (95 % CI of 3.01–16.1, *p* < 0.001) for less than passing performance on clerkship grades (Table 1) and on the clerkship NBME subject exams (Table 2), respectively. The multiple linear regression model of students’ performance in ICM and ICR, inclusive of the ICD NBME subject exam, explained 22 % of the variance of the average clerkship NBME subject exam score across all clerkships (Table 3). The ICD NBME subject exam score (*β* = 0.53, *p* < 0.0005) and ICR small-group points (*β* = 0.08, *p* = 0.014) were significant predictors of the dependent variable. As would be expected, the standardized coefficient, which represents the relative strength of the predictors in the model, of 0.53 for the ICD NBME subject exam indicates a fairly large effect size of 1.25 (95 % CI of 1.11–1.39) on the clerkship NBME subject exam score.

**Table 1:** The contingency table between at risk students at both Introduction to Clinical Medicine and Introduction to Clinical Reasoning courses and poor performance identified by average clerkship grades

		Average clerkship grades	
		Poor performance	No poor performance
At risk at both ICM/ICR	Observed count	5 (31.3 %)	11 (68.8 %)
	Expected count	0.9	15.1
Not at risk at both ICM/ICR	Observed count	21 (4.5 %)	447 (95.5 %)
	Expected count	25.1	442.9

Pearson *χ* ^2^ (1) = 21.80, *p* < 0.01. *ICD* Introduction to Clinical Diagnosis, *ICM* Introduction to Clinical Medicine

**Table 2:** The contingency table between at risk students identified by both Introduction to Clinical Medicine and Introduction to Clinical Reasoning courses and poor performance identified by average clerkship the National Board of Medical Examiners scores

		Average clerkship NBME scores	
		Poor performance	No poor performance
At risk at both ICM/ICR	Observed count	3 (18.8 %)	13 (81.3 %)
	Expected count	0.6	15.4
Not at risk at both ICM/ICR	Observed count	15 (3.2 %)	453 (96.8 %)
	Expected count	17.4	450.6

Pearson *χ* ^2^ (1) = 10.44, *p* < 0.05. *ICD* Introduction to Clinical Diagnosis, *ICM* Introduction to Clinical Medicine, *NBME* National Board of Medical Examiners

**Table 3:** Regression models of Introduction to Clinical Medicine and Introduction to Clinical Reasoning course performances to predict variance in average National Board of Medical Examiners subject exam scores across six clerkships

Explanatory variables	Standardized regression coefficient	*t*-statistic	*p*-value	R^2^ change
First year GPA	0.30	5.61	<0.0005	0.34
ICD NBME	0.53	13.59	<0.0005	0.22
ICR small-group points	0.08	10.97	0.014	
ICR exam points	−0.09	10.09	0.009	
ICM preceptor	−0.01	0.43	0.75	
ICM OSCE	0.05	1.51	0.14	

*GP* grade point average, *ICD* Introduction to Clinical Diagnosis, *ICM* Introduction to Clinical Medicine, *ICR* Introduction to Clinical Reasoning, *NBME* National Board of Medical Examiners, *OSCE* Objective Structured Clinical Examination

In terms of predicting the variance in average final grade across clerkships, this group of explanatory variables from the ICM and ICR courses accounted for 20.2 % of the variance beyond first-year grade point average (Table 4). All of the following explanatory variables were significant predictors, including ICD NBME subject exam score (*β* = 0.35, *p* < 0.0005), ICM preceptor evaluation (*β* = 0.11, *p* = 0.001), ICM OSCE score (*β* = 0.16,* p* < 0.0005), and ICR small-group points (*β* = 0.15, *p* < 0.0005). As opposed to exam scores, each of the predictors had relatively robust standardized regression coefficients translating into effects sizes of 0.75 for the ICD NBME exam, 0.22 for the ICM preceptor evaluation, 0.32 for the ICM OSCE score, and 0.30 for the ICR small-group points.

**Table 4:** Regression models of Introduction to Clinical Medicine and Introduction to Clinical Reasoning course performances to predict variance in average final grade across six clerkships

Explanatory variables	Standardized regression coefficient	*t*-statistic	*p*-value	R^2^ change
First year GPA	0.27	4.32	<0.0005	0.32
ICD NBME	0.35	4.84	<0.0005	0.20
ICM preceptor	0.11	3.87	0.001	
ICM OSCE	0.16	3.75	<0.0005	
ICR small-group points	0.15	4.34	<0.0005	
ICR exam points	0.04	4.42	0.351	

*GPA* grade point average, *ICD* Introduction to Clinical Diagnosis, *ICM* Introduction to Clinical Medicine, *ICR* Introduction to Clinical Reasoning, *NBME* National Board of Medical Examiners, *OSCE* Objective Structured Clinical Examination

## Discussion

Our study demonstrates several important findings regarding pre-clerkship clinical assessments. Students identified as at risk in both the ICM and ICR courses (defined as the bottom 13^th^ percentile) were found to be at significant risk for poor performance on the clerkship. Overall, the integration of clinical skills and clinical reasoning assessments from the pre-clerkship period demonstrate the ability to explain a significant amount of the variance in clerkship performance independent of previous academic abilities. As would be expected, the ICD NBME subject exam is a strong predictor for students’ future performance on clerkship NBME subject exams. However, clinical reasoning ability as measured by student small-group participation is also a significant predictor in the variance of future exam performance. Faculty observation of clinical skills and clinical reasoning using integrated assessments in combination with performance on the end of pre-clerkship OSCE are not just important predictors of student clerkship grades independent of previous academic abilities, but also demonstrate the ability to identify students who are likely to struggle on the clerkship. The fact that students who struggle in these pre-clerkship courses can both be readily identified and demonstrate significantly poorer performance during the clerkship has important implications for educators.

The ability to identify struggling students in the pre-clerkship period is an important aspect of developing and implementing appropriate remediation efforts in a timely fashion [6]. Arguably, using a combination of integrated clinical skills and clinical reasoning assessments to better understand and predict the variance in future clerkship performance has significant advantages over isolated pre-clerkship assessments that may not have adequate sensitivity to predict clerkship performance [7]. Not only can the use of these types of assessments predict overall performance, but specific deficits across a variety of domains (communication skills, professionalism, physical exam skills, or clinical reasoning) may be identified to provide remediation efforts tailored to a student’s individual needs in a controlled setting before moving on to the clerkship. Many schools utilize a clinical skills centre or simulation centre to deliver basic clinical skills and clinical reasoning curriculum in a standardized and relatively protected environment, which is a much more ideal setting for deliberate remediation efforts than allowing students to attempt remediation efforts in the clerkship [13]. In fact, based on the results of this study, our institutions simulation centre in coordination with pre-clerkship clinical skills faculty have coordinated efforts to identify and tailor the remediation of clinical skills and clinical reasoning for medical students before entering the clerkship period.

Our findings suggest that further integration of pre-clerkship courses in clinical skills and clinical reasoning could provide strong predictive power of students’ future clerkship performance, and should be confirmed in a prospective study. In fact, others have promoted the further integration of clinical skills with clinical reasoning where the student can learn both content skills related to accomplishing a basic patient history and physical exam simultaneous with clinical reasoning within the context of a clinical setting [2]. Pre-clerkship curriculum should develop integrated clinical experiences to develop habits of inquiry and opportunities for the application of medical knowledge and clinical skills (patient history and exam) to clinical reasoning [18]. Based upon our findings, we have continued to integrate the ICM and ICR courses, and now have five integrated clinical skill sessions in the pre-clerkship period that have small-group clinical reasoning sessions immediately after standardized patient encounters identical to the context of an ambulatory clerkship setting. Preliminary data indicate that students who perform in the bottom 13^th^ percentile on these five integrated sessions have a relative risk of 2.02 (95 % CI 1.33–3.06; *p* < 0.001) of being identified as struggling on the internal medicine clerkship. In addition, we have implemented the use of longitudinal faculty during the pre-clerkship training, which may further improve our ability to identify students at risk for struggling in the clerkship, and provide opportunities for early intervention.

Ultimately, the use of clinical skills centres providing an integrated clinical skills and clinical reasoning curriculum under a controlled environment with input from clerkship directors may be the most beneficial for students, and potentially promote the longitudinal development and vertical integration of these foundational abilities [13].

Our study had several limitations. First, this is a single institution study, and although we had a large number of students in the analysis, our findings may not be generalizable to all other medical schools. Second, we used first year medical school grade point average as a control variable for baseline academic ability in our regression models. Although first year grade point average strongly predicts the outcome measures on the clerkship in our study, it is possible that other academic outcomes from the second year of medical school could have been even more predictive, and lowered the overall predictive power of the ICM and ICR assessments. Additionally, the ICD NBME exam was predictive of clerkship outcomes, especially for future NBME subject exams. However, the standardized regression coefficients for the integrated ICM and ICR outcomes indicate strong contributions of these variables to the overall model, and there is significant construct validity to relate performance on clinical skills and clinical reasoning assessments to these same measures on the clerkship. Of note, the three year longitudinal nature of our study is an important strength, as this represents a fairly large group of students moving through a stable, integrated pre-clerkship curriculum for clinical skills and clinical reasoning.

## Conclusion

Our study demonstrates the ability of integrated clinical skills and clinical reasoning assessments to identify at risk students, and explain the variance of students’ future performance in the clerkship, emphasizing the importance of an integrated pre-clerkship curriculum to foster opportunities for early identification and individualized remediation of students before reaching the clerkships. There could be a significant advantage to accurately identify a group of learners struggling in basic clinical skills before entering the clerkship environment, allowing for timely remediation of these critical skills under direct observation in a controlled environment.
